# Supplementary material for: Mass production of 2D materials by intermediate-assisted grinding exfoliation
Source: Natl Sci Rev. 2019 Oct 21;7(2):324–32. doi: 10.1093/nsr/nwz156 (PMC8288955; doi:10.1093/nsr/nwz156)
Supplement: nwz156_Supplemental_File [file nwz156_supplemental_file.docx]

Supplementary Information for *National Science Review*

**Mass Production of Two-Dimensional Materials by Intermediate-Assisted Grinding Exfoliation**

Chi Zhang^1+^, Junyang Tan^1+^, Yikun Pan^1^, Xingke Cai^1^, Xiaolong Zou^1^, Hui-Ming Cheng^1,2,3^* and Bilu Liu^1^*

^1^Shenzhen Geim Graphene Center (SGC), Tsinghua-Berkeley Shenzhen Institute (TBSI) & Tsinghua Shenzhen International Graduate School (TSIGS), Tsinghua University, Shenzhen 518055, P. R. China.

^2^Shenyang National Laboratory for Materials Science, Institute of Metal Research, Chinese Academy of Sciences, Shenyang 110016, P. R. China.

^3^Advanced Technology Institute (ATI), University of Surrey, Guildford, Surrey GU2 7XH, UK.

^+^These authors contributed equally.

*E-mail: [bilu.liu@sz.tsinghua.edu.cn](mailto:bilu.liu@sz.tsinghua.edu.cn) (Bilu Liu), [hmcheng@sz.tsinghua.edu.cn](mailto:hmcheng@sz.tsinghua.edu.cn) (Hui-Ming Cheng)

1. **Characterization and analysis**

**1.1 Spectroscopic characterization of the iMAGE exfoliated 2D h-BN**

XPS analysis of the B1s and N1s peaks of 2D h-BN indicates that there is no noticeable broadening compared to raw bulk h-BN, confirming the high quality of the material obtained. FTIR analysis of the exfoliated 2D and as-received raw bulk h-BN shows that that a negligible number of functional groups had been grafted onto the exfoliated 2D h-BN during the iMAGE process, consistent with XPS results. The absorption peaks in FTIR are mainly ascribed to in-plane B-N stretching at 1367 cm^-1^ and out-of-plane B-N-B bending at 807 cm^-1^, with no signals from other functional groups. The forces involved in our process are much smaller than in high energy ball milling and other reported methods for the scalable exfoliation of layer materials.[1,2] The strong B-N vibration mode (E_2g_) at around 1366 cm^-1^ in the Raman spectra for the exfoliated 2D h-BN also indicates its high quality.[3]

**1.2 Calculation of the exfoliation yield**

The as-exfoliated 2D material dispersion was centrifuged at 1500 rpm for 30 minutes, and then vacuum filtered onto an alumina membrane with a pore size of 20 nm. After vacuum drying (80℃ for 12 hours), the powder sample obtained was weighed and used to calculate the exfoliation yield. XPS results showed that a small amount of SiC remained in the supernatant (~4.4 wt%, see Figure S7), and this was subtracted and not included in the calculation of the yield.

Exfoliation yield was also calculated from UV-vis-NIR absorption spectra as the ratio of the absorption value at 300 nm after centrifugation to that before centrifugation.

**1.3 Calculation of the extinction coefficient**

Dried 2D h-BN powder was added to isopropanol with an initial concentration of 0.2 mg mL^-1^, which was then diluted with isopropanol to concentrations ranging from 0.005 to 0.2 mg mL^-1^. The UV-vis-NIR absorption spectra of the suspension in the 200-800 nm wavelength range were recorded using a quartz cell with an optical length (*l)* of 10 mm. Based on the Beer-Lambert law, *A_λ_*/*l*=α*_λ_C,* where *A_λ_* is the absorbance at a specific wavelength, α is the extinction coefficient, and *C* is the concentration of the 2D material, the extinction coefficient α*_λ_* at a specific wavelength was obtained by fitting *A*/*l* to *C*.

**1.4 Calculations of the average friction force produced by SiC force intermediates**

The diameter of the ram (*d*_ram_) of the RM200 equipment is 66.8 mm and the weight ratio of the bulk h-BN to the SiC intermediate (*m*_SiC_)/(*m*_h-BN_) is 1/4. The gravimetric densities of SiC (*ρ*_SiC_) and h-BN (*ρ*_h-BN_) are 3.2 and 2.27 g cm^-3^, respectively. We treat the two materials as spherical to simplify the calculation, which gives the ratio of contact area for the intermediate material/ram to that for the the layer material/ram, which depends on the cross-sectional area of the two different materials, as [(*m*_SiC_/*ρ*_SiC_)/(*m_h_*_-BN_/*ρ*_h-BN_)]^2/3^. The ratio of the contact area for SiC particles/ram is (*m*_SiC_/*ρ*_SiC_)^2/3^/[(*m*_SiC_/*ρ*_SiC_)^2/3^ + (*m*_h-BN_/*ρ*_h-BN_)^2/3^]. The numbers of intermedies, *n*, in each dashed box of Fig. 1a, are given by *n* = (*d*_ram_/*d*_SiC_)^2^ (*m*_SiC_/*ρ*_SiC_)^2/3^/[(*m*_SiC_/*ρ*_SiC_)^2/3^ + (*m*_h-BN_/*ρ*_h-BN_)^2/3^], where *d*_SiC_ is the diameter of the SiC intermediate. The average force loaded onto each intermediate particle is *f*_i_=*F*_c_/*n*, where the macroscopic compressive force *F*_c_ is around 100 Newtons. The average frictional force loaded from each intermediate particle *f*_fi_ = *μf*_i_ = *μF*_c_/*n*, where the sliding coefficient of friction for the h-BN is around 0.2.[4] The values of *n*, *f*_i_ and *f*_fi_ for different intermediate particle sizes *D* and different weight ratios of the intermediate to the layer material *N* are shown in Table S4 and the following table.

| Weight ratio *N*  (SiC:h-BN) | *n* | *f*_fi_ (μN) |
| --- | --- | --- |
| 0.5 | 1.52×10^5^ | 131.6 |
| 1 | 2.02×10^5^ | 99.0 |
| 1.5 | 2.32×10^5^ | 86.2 |
| 2 | 2.54×10^5^ | 78.7 |
| 3 | 2.84×10^5^ | 70.4 |
| 4 | 3.04×10^5^ | 65.8 |

Based on the above analysis, we estimate that when the diameter of the ram is increased to 2 meters, which is feasible, the contact area of the ram with the grinding mixture would be increased 900×. If the macroscopic compressive force and the total weight of the mixture (SiC and bulk h-BN) was also increased 900×, the production rate of 2D h-BN could reach 1.55 kg h^-1^ based on the production rate achieved using our present instrument, which would correspond to an annual production capability of >10 tons per instrument.


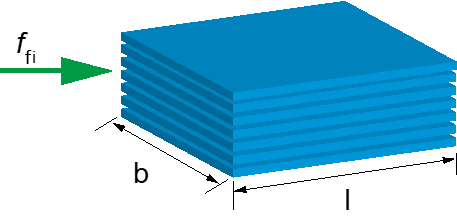


**Supplementary Figure S1. Scheme of the exfoliation of layer materials with width (b) and length (l) by a friction force *f*_fi_.** In order to have a successful exfoliation, it should satisfy *f*_fi_*l* > *blE*_e_, i.e., *f*_fi_ > *bE*_e_, as discussed in the main text. Note here *b* is the width of the layered materials, and the unit of *bE*_e_ is (m)*(J*m^-2^) = N.


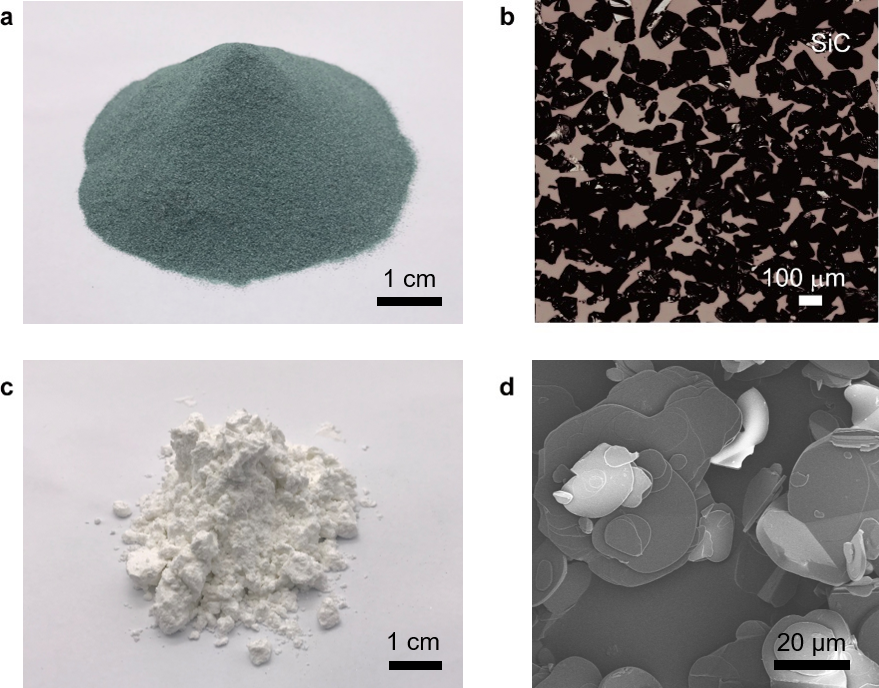


**Supplementary Figure S2. Characterization of SiC force intermediate and raw bulk h-BN. a-b,** Photographs and optical microscope images of SiC intermediates (150 mesh) with a green color. **c-d,** Optical and SEM images of the h-BN, which has an average thickness > 1 μm and a lateral size > 30 μm.


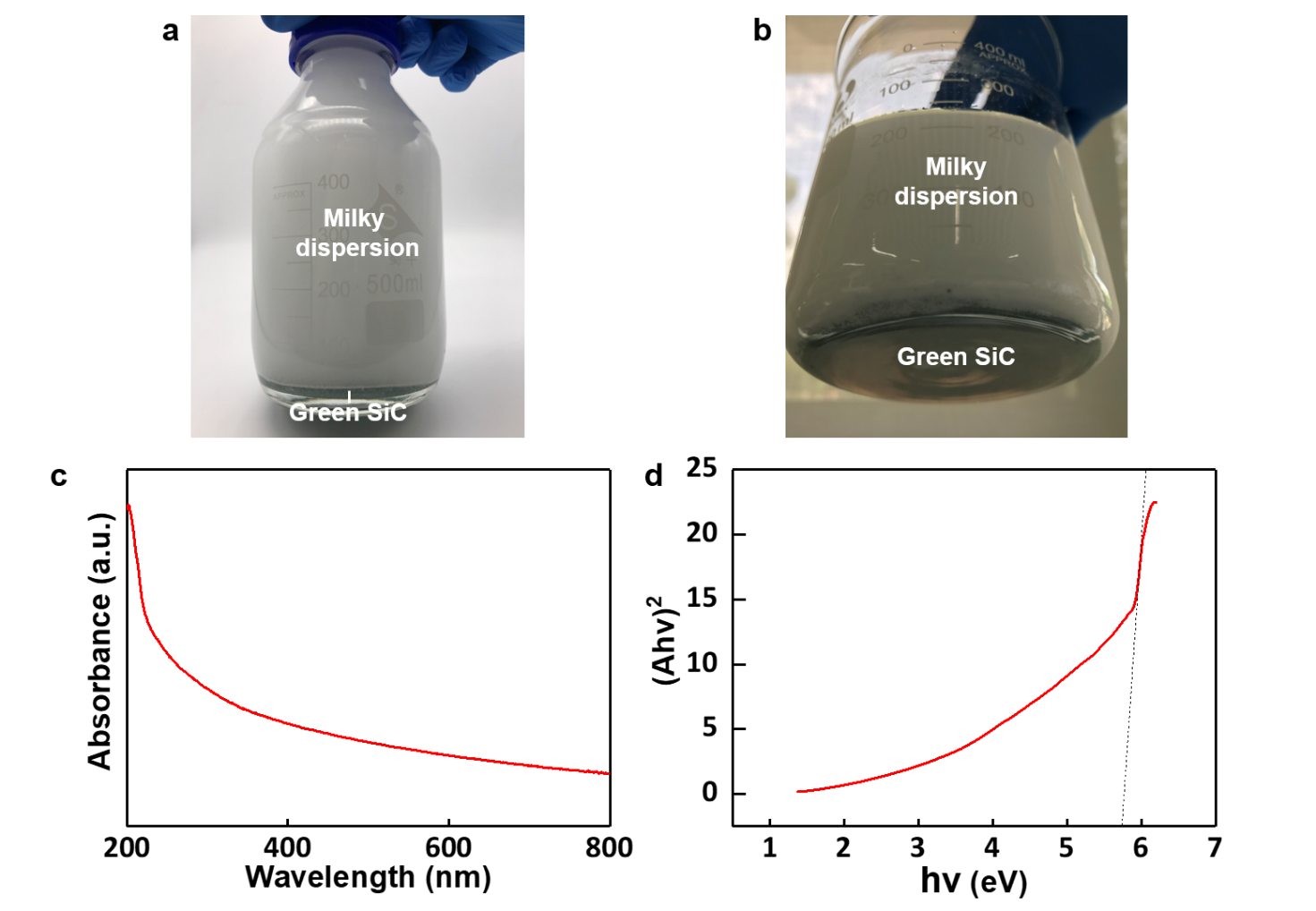


**Supplementary Figure S3. Separation of exfoliated 2D h-BN from SiC, and UV-vis-NIR absorption spectroscopy of the supernatant.** **a-b,** The ground h-BN/SiC mixture dispersed in DI water. The exfoliated 2D h-BN flakes are stably dispersed in the dispersion, while the un-exfoliated thick h-BN and green SiC precipitate at the bottom of the flask. **c,** A typical UV-vis-NIR absorption spectrum of the supernatant, which contains 2D h-BN. **d,** (A*h*ν)^2^ plotted versus *h*ν, where A is the absorbance and *h*ν is the photon energy, from which the bandgap of BN is calculated to be 5.8 eV, based on the Kubelka-Munk equation.


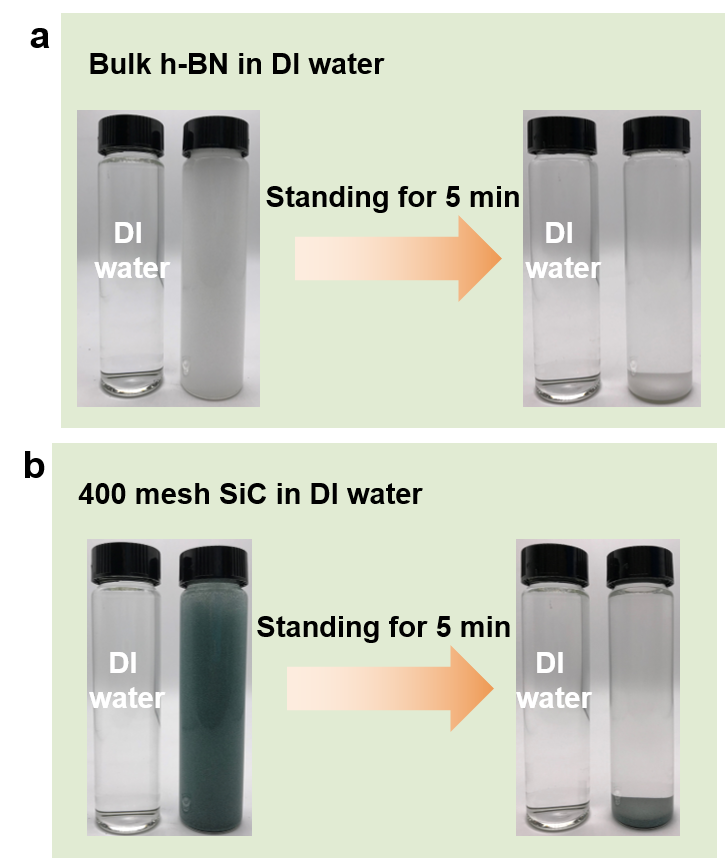


**Supplementary Figure S4. Precipitation of bulk h-BN and SiC in DI water upon standing, showing that they are not stably dispersed in DI water.** These results indicate that the material in the milky solution in **Fig. S2a**-**b** is exfoliated 2D h-BN.


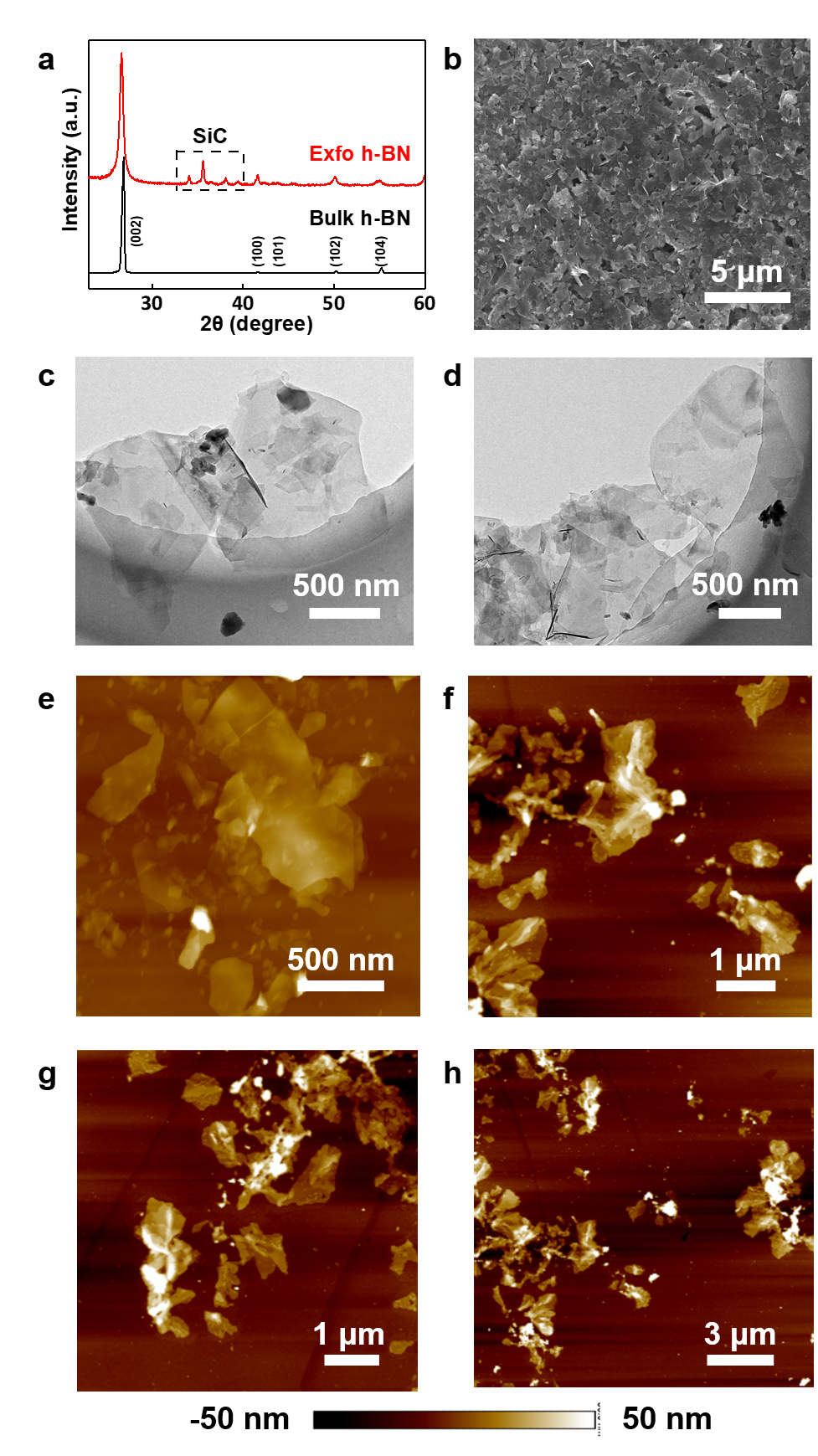


**Supplementary Figure S5. More characterization of exfoliated 2D h-BN. a.** XRD patterns of the exfoliated and bulk h-BN. **b.** SEM image, and **c, d.** TEM images. **e-h.** AFM images of the exfoliated 2D h-BN.


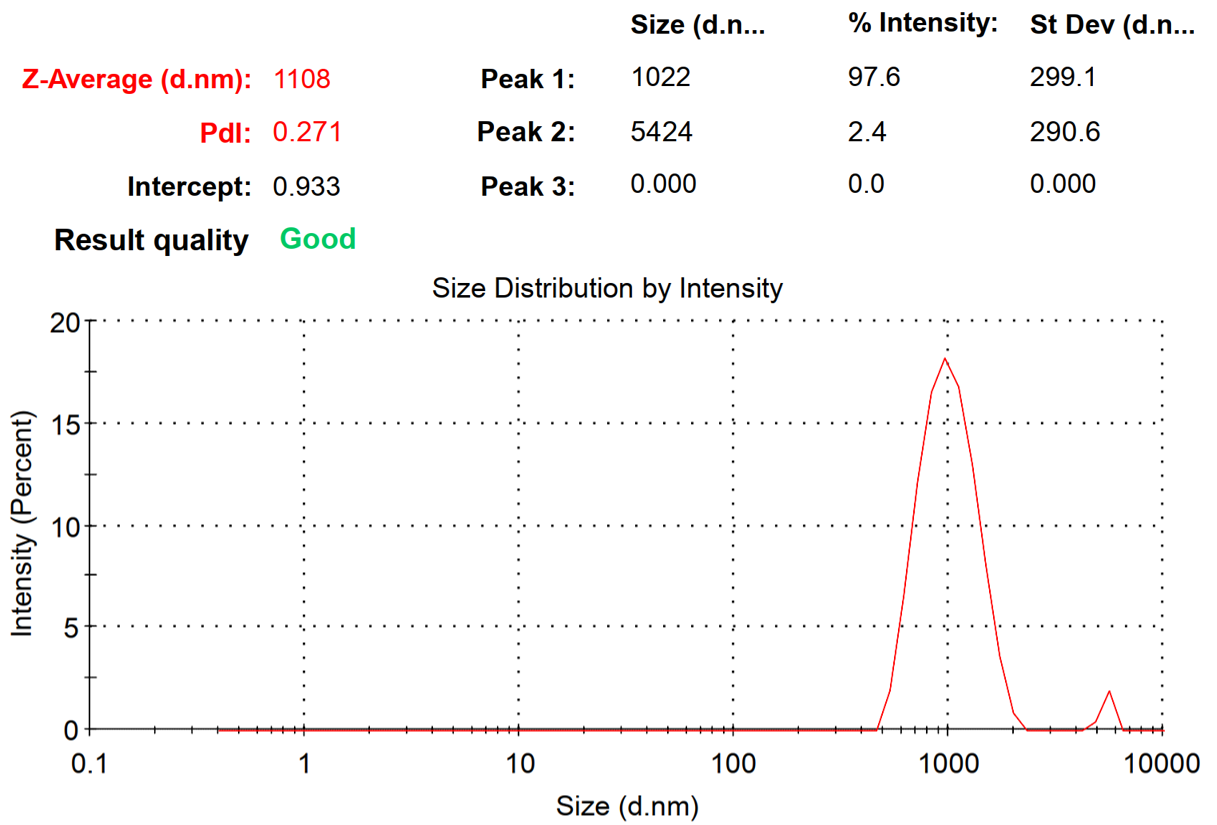


**Supplementary Figure S6. Dynamic light scattering (DLS) measurements of exfoliated 2D h-BN.** The results show that the average size of the as-prepared 2D h-BN is 1.1 μm, consistent with the AFM and TEM results.


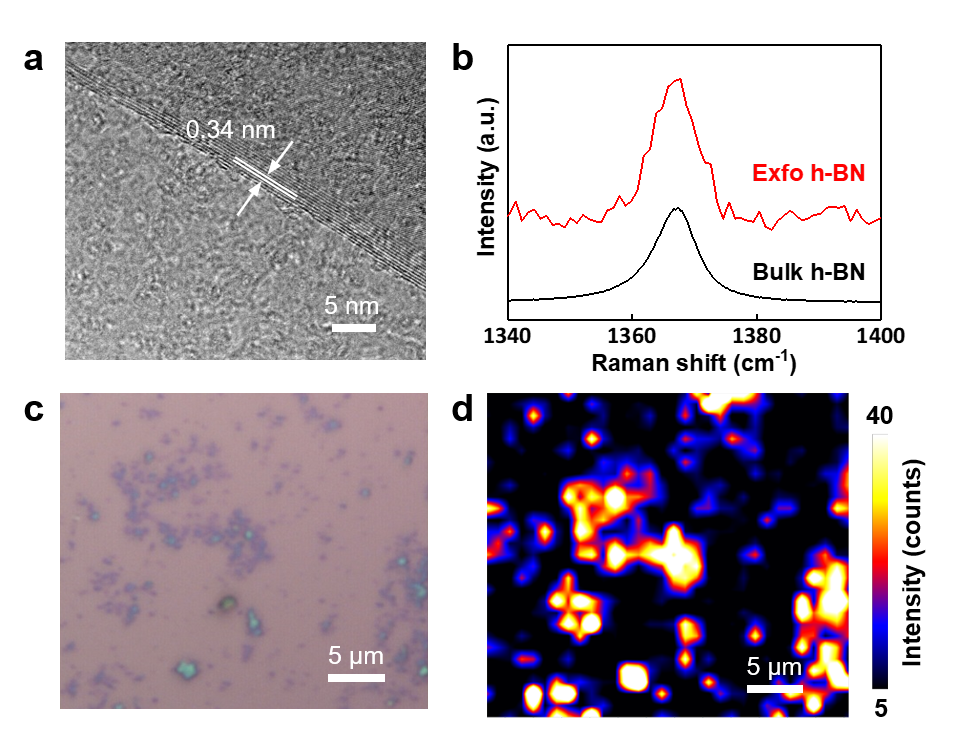


**Supplementary Figure S7. Further evidence of the high quality of the iMAGE exfoliated 2D h-BN. a,** A TEM image of the edges of exfoliated 2D h-BN. The straight edges with an interlayer spacing of 0.34 nm indicate its high quality. **b,** Raman spectra of the exfoliated 2D h-BN in **c** and bulk h-BN, showing the same E_2g_ peak at 1366 cm^-1^, which originates from the B-N vibrational mode in h-BN. **c-d,** Optical image of 2D h-BN dispersed on a SiO_2_/Si substrate and the corresponding Raman map of the B-N vibrational mode in h-BN, showing that the flakes are 2D h-BN.


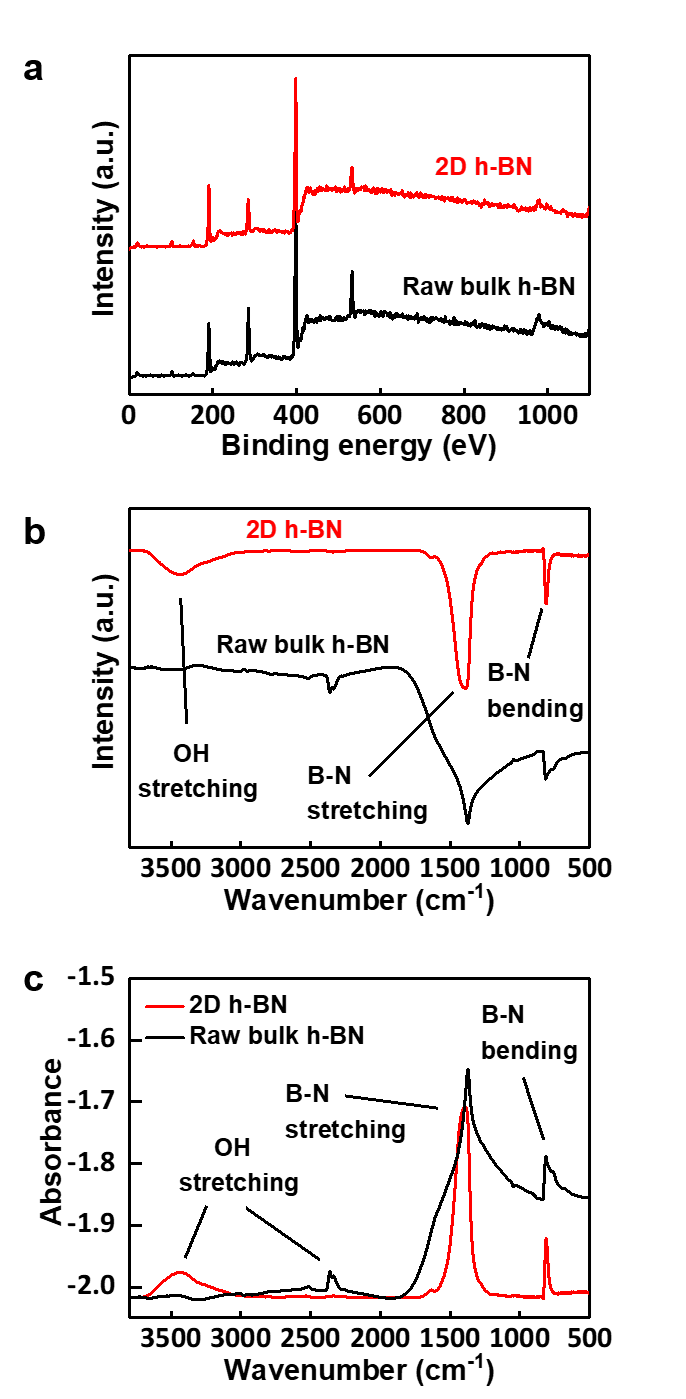


**Supplementary Figure S8. XPS and FTIR spectra of the exfoliated 2D h-BN and bulk h-BN (Enlarged view of Fig. 1i-j).** The results show that the iMAGE-produced 2D h-BN is pristine h-BN without functional groups.


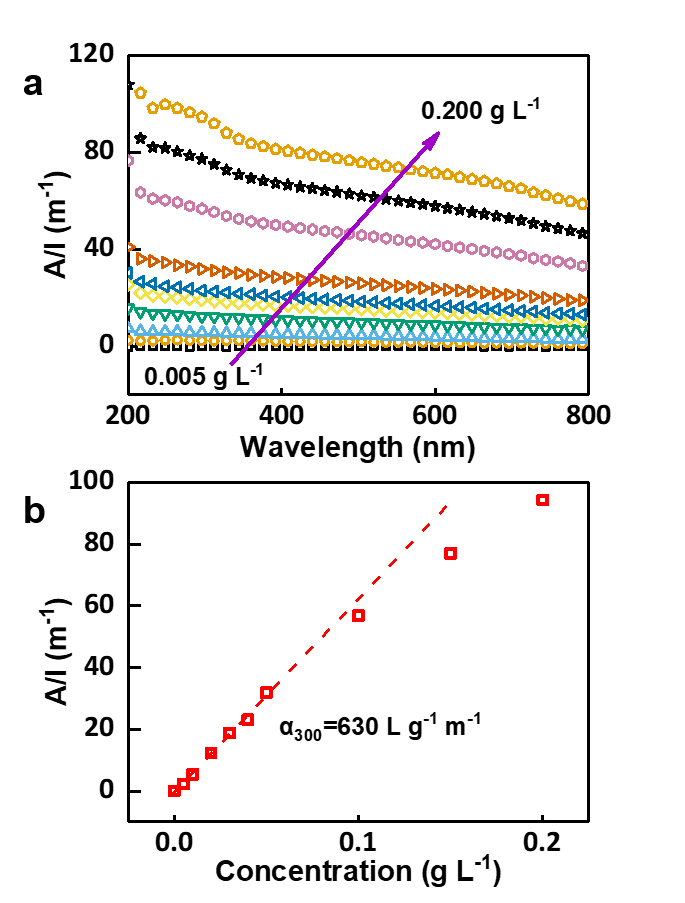


**Supplementary Figure S9. Calculations of the extinction coefficient of 2D h-BN according to the Beer-Lambert Law. a,** Absorption spectra of 2D h-BN dispersions with different concentrations. **b,** Beer-Lambert plots for 2D h-BN by fitting the absorbance values *A*_300_/*l* at different concentrations *C.* Based on the law *α*_300_*=(A*_300_*/l)/C* [5], we obtained an extinction coefficient of 630 L g^-1^ m^-1^ for 2D h-BN. Note that all spectra were collected by a UV-vis-NIR spectrophotometer with an integrating sphere accessory.


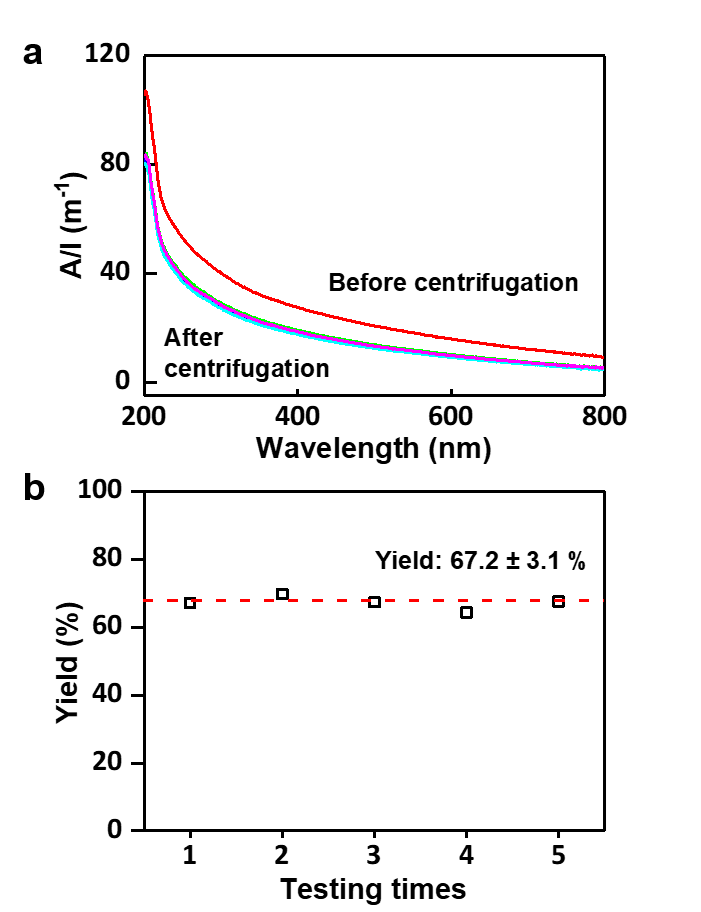


**Supplementary Figure S10. Calculations of the yield of 2D h-BN according to absorption spectra. a,** The absorption spectra of 2D h-BN dispersions before/after centrifugation. **b,** Yields calculated by the absorbance values at 300 nm (*α*_300after_/*α*_300before_).


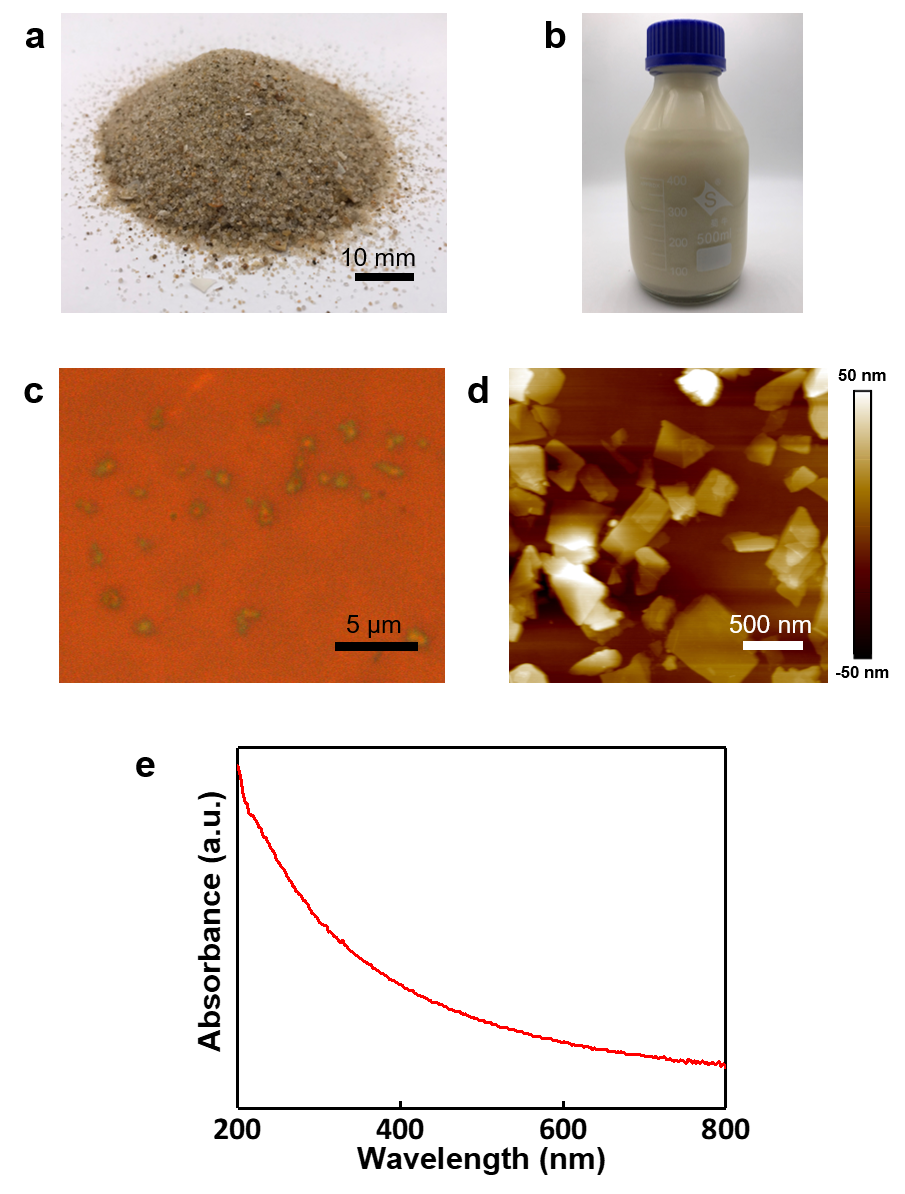


**Supplementary Figure S11. Exfoliation of h-BN using cheap sea sands as the force intermediate in iMAGE technology. a-b,** Photographs of sea sand and an exfoliated 2D h-BN dispersion. **c,** Optical microscope image, **d,** AFM image and **e,** absorption spectra of the as-prepared 2D h-BN. As the sea sands were collected from a beach directly without any further washing, there were some impurities such as shells and other seaside items that have densities close to water. When applying these sea sands as intermediate particles, longer standing time or centrifugation is needed to separate those impurities from the 2D materials dispersions, and similar to the case of SiC. UV-vis-NIR spectra, AFM and optical microscope images reveal the successful exfoliation of 2D h-BN.


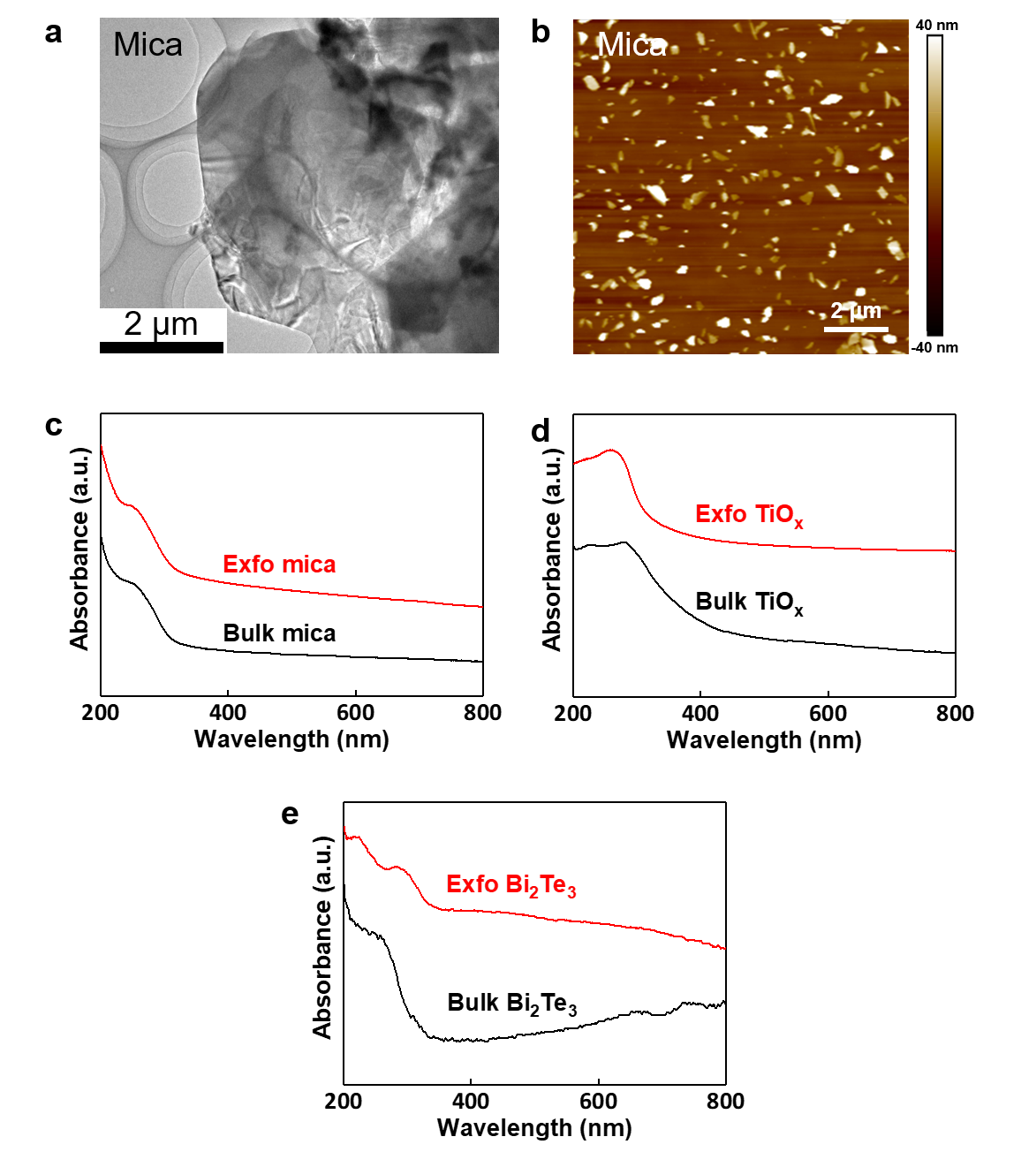


**Supplementary Figure S12. Use of the iMAGE technology to exfoliate mica, TiO_x_ and Bi_2_Te_3_. a,** TEM image. **b,** AFM image. **c-e,** UV-vis-NIR absorption spectra. For UV-vis-NIR spectra of as-exfoliated 2D mica and TiO_x_, the unchanged characteristic absorption bands show the high quality of these final products [6,7] The absorption peak appeared at 265 nm reveals the existence of few layer 2D Bi_2_Te_3_ [8].


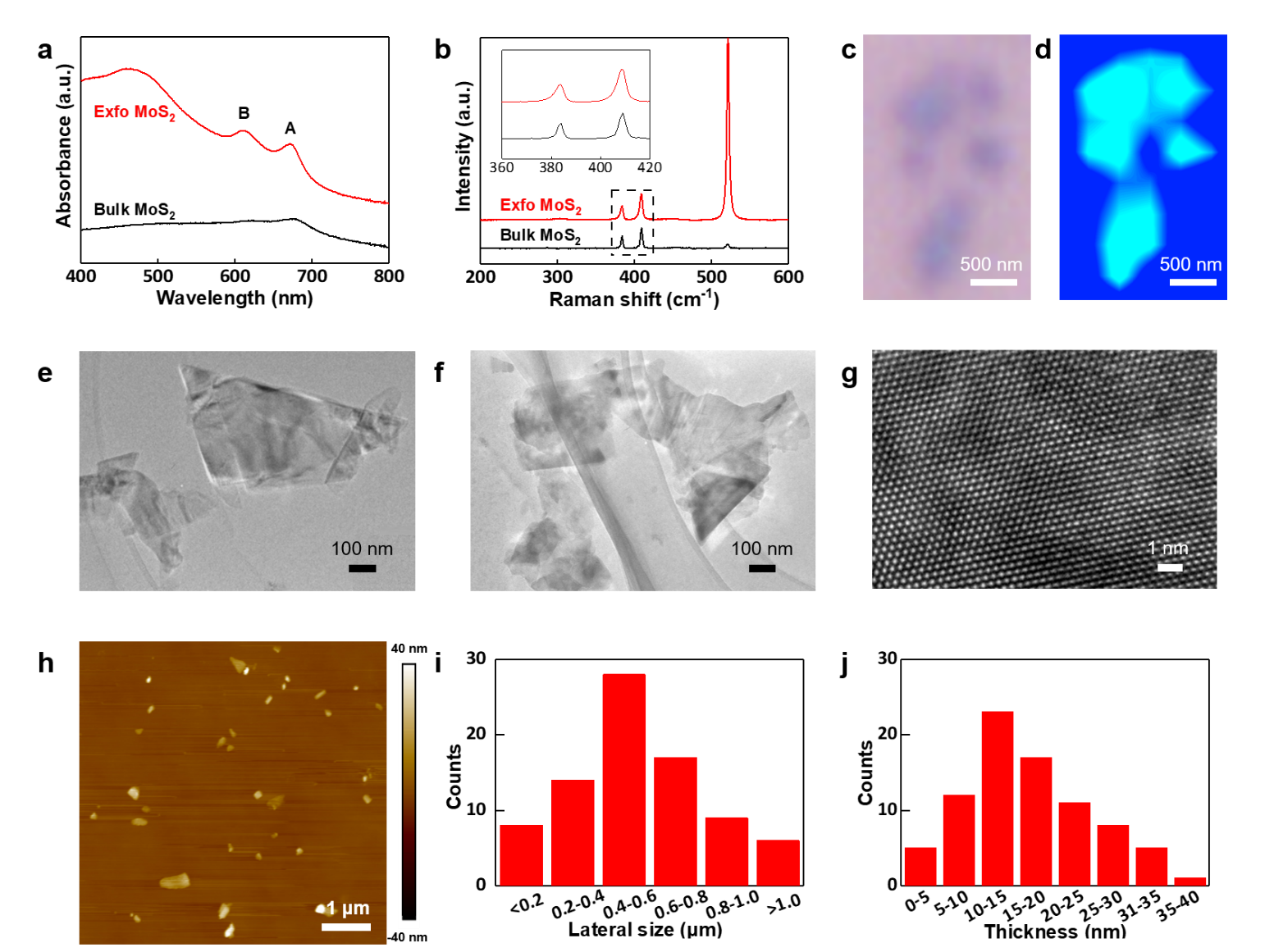


**Supplementary Figure S13. More characterization of exfoliated 2D MoS_2_. a,** UV-vis-NIR absorption spectra of exfoliated 2D MoS_2_ dispersed in DI water. **b,** Raman spectra. **c-d,** Optical image of 2D MoS_2_ dispersed on a SiO_2_/Si substrate and the Raman map of the Mo-S vibrational mode at 409 cm^-1^. **e-g,** TEM and HRTEM images. **h-j,** AFM image, statistical analysis of the lateral size and thickness of the as-prepared 2D MoS_2_.

**
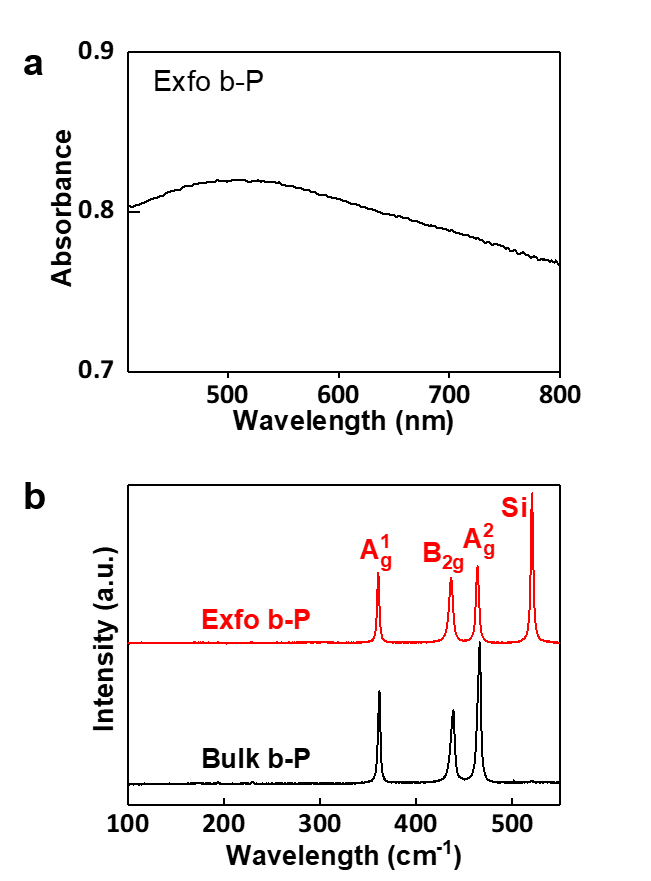
**

**Supplementary Figure S14.** **Use of the iMAGE technology to exfoliate b-P. a,** UV-vis-NIR absorption spectrum (Enlarged view of Fig. 3b) and **b,** Raman spectrum of exfoliated 2D b-P. UV-vis-NIR absorption spectrum shows a broad absorption peak around 520 nm as the wavelength range used has a much higher energy than the bandgap of b-P, which is similar to the previous reported results.[9] Raman spectroscopy of b-P shows that the A_g_^1^/A_g_^2^ ratios of exfoliated b-P and the bulk material were 0.88 and 0.68, respectively, which are both larger than the oxidation criterion value (0.6) and indicates that the 2D b-P flakes obtained were not oxidized after iMAGE exfoliation.[10]


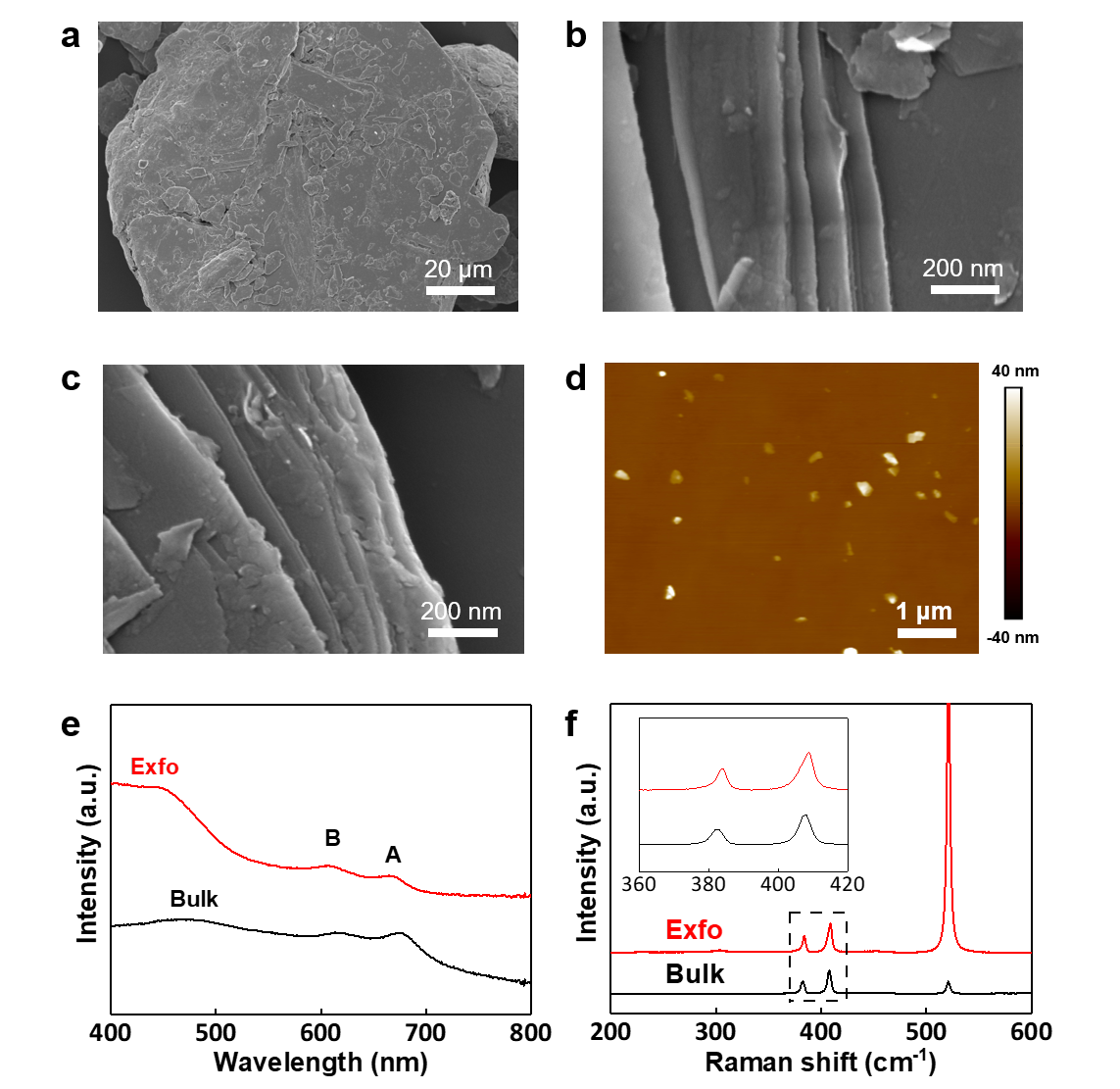


**Supplementary Figure S15. Scaling up demonstration of production of 2D MoS_2_ from a cheap and abundant natural resource, molybdenite concentrate. a-c,** SEM images of the molybdenite concentrates, showing the layer structure. **d,** AFM image. **e,** UV-vis-NIR absorption spectra. **f,** Raman spectra of the exfoliated 2D molybdenite concentrate dispersed in DI water.

**Supplementary Table S1. Comparison of 2D h-BN prepared by different methods**

| **Methods** | **Starting raw material (mg)** | **Production**  **rate**  **(mg h^-1^)** | **Quality** | **Yield** | **Lateral size**  **(μm)** | **Thickness**  **(nm)** | **Concentration**  **in dispersion**  **(mg mL^-1^)** | **Energy consumption**  **(J g^-1^)** |
| --- | --- | --- | --- | --- | --- | --- | --- | --- |
| Micromechanical cleavage [11,12] | N.A. | N.A. | Very high | N.A. | 1-100 | 0.6-3 | N.A. | N.A. |
| Electrochemical intercalation [13] | 1-100 | 10^-2^ | High | 90% | 0.5-0.7 | 2-10 | Tunable | 2.52×10^9^ |
| Ball milling in  solid medium [1] | 1000 | ～41.5 | NH_2_-functionalized | 83% | <0.1 | 2.5 | Tunable  (up to 30) | 7.04×10^7^ |
| Ball milling in liquid medium [14] | 2000 | 15 | OH-functionalized | 18% | 1.5 | 2-3 | Tunable  (0.1-0.4） | 3.6×10^8^ |
| Low energy ball milling [15] | 500 | 22.3 | High | 67% | 0.1-1 | 1-4 | Tunable | 2.1×10^8^ |
| Sonication [5] | 30-150 | 2.5Х10^-2^ | High | 2 % | 0.3-1 | 1-5 | 0.06 | 1.96×10^9^ |
| Sonication [16]  (based on graphene) | 2310 | 1.83 | High | 36.4% | 1 | 1-5 | 1.2 | 4.54×10^8^ |
| Sonication-assisted hydrolysis [17] | 20 | 0.125 | OH functionalized | 5% | 0.01 -1 | 1-10 | 0.1 | 1.44×10^9^ |
| Shearing force assisted liquid exfoliation (based on graphene) [2] | 3×10^7^ | 5300 | High | 0.1% | 0.3-1 | 1-5 | 0.07 | 2.04×10^7^ |
| Turbulence-assisted exfoliation [18] (based on MoS_2_) | 50,000 | 78 | High | 1% | 0.04-0.2 | 1-10 | 0.4 | 8.77×10^7^ |
| Molten hydroxides assisted exfoliation [19] | 248 | 0.235 | Curled edge but good crystallinity | 0.191% | 0.1-5 | 1-6 | - | 1.30×10^7^ |
| **iMAGE**  **(This work)** | **2000** | **~300** | **High** | **67%** | **1.2**  **(average)** | **1-5** | **Tunable**  **(up to 15)** | **3.01×10^6^** |

**Supplementary Table S2. Relationship between intermediate size (*D*), the population (*n*) and the average compressive force and frictional force produced by each particle (*f*_fi_) by fixing the total weight of the intermediate used**

| Mesh | Intermediary size  *D* (μm) | *n* | *f*_fi_ (μN) | *f*_i_ (μN) |
| --- | --- | --- | --- | --- |
| 26 | 570.5 | 1.37×10^4^ | 1460 | 7300 |
| 60 | 246.7 | 7.33×10^4^ | 273 | 1365 |
| 150 | 98.9 | 4.56×10^5^ | 65.8 | 329 |
| 400 | 37 | 3.26×10^6^ | 6.13 | 30.65 |
| 800 | 18.5 | 1.30×10^7^ | 1.54 | 7.7 |
| 2000 | 7.4 | 8.15×10^7^ | 0.245 | 1.225 |

**Supplementary Table S3. Comparison of the ball milling and iMAGE methods based on the surface area of the intermediates and their population**

|  | Ball milling [11] | This work (iMAGE) |
| --- | --- | --- |
| Intermediary | Steel balls | SiC particles |
| Weight of intermediates (g) | 100 | 8 |
| Diameter of Intermediates (mm) | 8 | 0.1 |
| Weight density (g/cm^3^) | 7.9 | 3.2 |
| Volume (cm^3^) | 12.6 | 2.5 |
| Surface area (cm^2^) | 96.5 | 187.5 |
| Population of the intermediate used for exfoliation | 50 | 5.97×10^5^ |

**Supplementary Table S4. Calculations of the theoretical exfoliation energy of different layer materials**

|  | *a* (nm) | Unit area  (nm^2^) | Binding energy  per atom (meV) | Binding energy  (J m^-2^) | Cleavage energy  (J m^-2^) | Exfoliation energy  (J m^-2^) |
| --- | --- | --- | --- | --- | --- | --- |
| Graphene | 0.247 | 0.0524 | 24 [20] | 0.1466 | 0.1725 | 0.2029 |
| h-BN | 0.251 | 0.0546 | 26 [21] | 0.1524 | 0.1793 | 0.2109 |
| MoS_2_ | 0.323 | 0.0903 | 60 [21] | 0.3189 | 0.3752 | 0.4414 |
| WSe_2_ | 0.328^35^ | 0.0932 | 62.1 | 0.32 [22] | 0.3765 | 0.4429 |

**Supplementary Table S4. Continued**

|  | Molecular weight  (g mol^-1^) | Area density  (g m^-2^) | Weight of a 30×30 μm^2^ monolayer  (g) | Number of layers  per 1 g | Exfoliation energy of one layer from the bulk material  (J) | Total exfoliation energy for 1 g of material to become a monolayer  (J g^-1^) |
| --- | --- | --- | --- | --- | --- | --- |
| Graphene | 12 | 0.761×10^-3^ | 6.85×10^-13^ | 1.46×10^12^ | 1.826×10^-10^ | 266.6 |
| h-BN | 24.8 | 0.755×10^-3^ | 6.80×10^-13^ | 1.47×10^12^ | 1.898×10^-10^ | 279.0 |
| MoS_2_ | 160 | 2.943×10^-3^ | 2.65×10^-12^ | 3.77×10^11^ | 3.973×10^-10^ | 149.8 |
| WSe_2_ | 341.7 | 6.090×10^-3^ | 5.48×10^-12^ | 1.82×10^11^ | 3.986×10^-10^ | 72.5 |

**REFERENCES**

1. Lei, W. *et al.* Boron nitride colloidal solutions, ultralight aerogels and freestanding membranes through one-step exfoliation and functionalization. *Nat. Commun.* **6**, 8849 (2015).

2. Paton, K. R. *et al.* Scalable production of large quantities of defect-free few-layer graphene by shear exfoliation in liquids. *Nat. Mater.* **13**, 624-630 (2014).

3. Sainsbury, T. *et al.* Oxygen radical functionalization of boron nitride nanosheets. *J. Am. Chem. Soc.* **134**, 18758 (2012).

4. Scharf, T. W. & Prasad, S. V. Solid lubricants: a review. *J Mater Sci* **48**, 511-531 (2013).

5. Coleman, J. N. *et al.* Two-dimensional nanosheets produced by liquid exfoliation of layered materials. *Science* **331**, 568-571 (2011).

6. Osada, M. et al. Controlled doping of semiconducting titania nanosheets for tailored spinelectronic materials. *Nanoscale* **6**, 14227-14236 (2014).

7. Pan, X. F. et al. Transforming ground mica into high-performance biomimetic polymeric mica film. *Nat. Commun.* **9**, 2974 (2018).

8. Srivastava, P. & Singh, K. Low temperature reduction route to synthesise bismuth telluride (Bi_2_Te_3_) nanoparticles: structural and optical studies. *J. Exp. Nanosci.* **9**, 1064-1074 (2014).

9. Woomer, A. H. *et al.* Phosphorene: synthesis, scale-up, and quantitative optical spectroscopy. *ACS Nano.* **9**, 8869 (2015).

10. Hanlon, D. et al. Liquid exfoliation of solvent-stabilized few-layer black phosphorus for applications beyond electronics. *Nat. Commun.* **6**, 8563 (2015).

11. Novoselov, K. S. *et al.* Electric field effect in atomically thin carbon films. *Science* **306**, 666-669 (2004).

12. Novoselov, K. S. *et al.* Two-dimensional atomic crystals. *PNAS* **102**, 10451-10453 (2005).

13. Zeng, Z. *et al.* An effective method for the fabrication of few-layer-thick inorganic nanosheets. *Angew. Chem. Int. Ed.* **51**, 9052-9056 (2012).

14. Lee, D. *et al.* Scalable exfoliation process for highly soluble boron nitride nanoplatelets by hydroxide-assisted ball milling. *Nano Lett.* **15**, 1238-1244 (2015).

15. Li, L. H. *et al.* Large-scale mechanical peeling of boron nitride nanosheets by low-energy ball milling. *J. Mater. Chem.* **21**, 11862-11866 (2011).

16. Khan, U., O'Neill, A., Lotya, M., De, S. & Coleman, J. N. High concentration solvent exfoliation of graphene. *Small* **6**, 864-871 (2010).

17. Lin, Y. *et al.* Aqueous dispersions of few-layered and monolayered hexagonal boron nitride nanosheets from sonication-assisted hydrolysis: critical role of water. *J. Phys. Chem. C* **115**, 2679-2685 (2011).

18. Varrla, E. *et al.* Large-scale production of size-controlled MoS_2_ nanosheets by shear exfoliation. *Chem. Mater.* **27**, 1129-1139 (2015).

19. Li, X. *et al.* Exfoliation of hexagonal boron nitride by molten hydroxides. *Adv. Mater.* **25**, 2200-2204 (2013).

20. Rydberg, H. *et al.* Van der Waals density functional for layered structures. *Phys. Rev. Lett.* **91**, 126402 (2003).

21. Björkman, T. Testing several recent van der Waals density functionals for layered structures. *J. Chem. Phys.* **141**, 246401 (2014).

22. Björkman, T., Gulans, A., Krasheninnikov, A. V. & Nieminen, R. M. Van der Waals bonding in layered compounds from advanced density-functional first-principles calculations. *Phys. Rev. Lett.* **108**, 235502 (2012).
